# Science-Based Risk Assessment for the Categorization of Visual Inspection Defects of Sterile Dosage Forms

**DOI:** 10.3390/pharmaceutics17091121

**Published:** 2025-08-27

**Authors:** Hanns-Christian Mahler, Emilien Folzer, Ragunath Ananthavettivelu, Jonas Koehler, Morgana Ferrari, Andrea Allmendinger

**Affiliations:** 1 ten23 health AG, Mattenstrasse 22, 4057 Basel, Switzerland; 2 ten23 health Valais AG, Rottenstrasse 5, 3930 Visp, Switzerland; 3 Department of Pharmaceutics, Institute of Pharmaceutical Sciences, University of Freiburg, Sonnenstr. 5, 79104 Freiburg im Breisgau, Germany

**Keywords:** visual inspection, parenteral drug products, risk assessment, defect classification, quality control, FMEA, sterile dosage forms, visible particles

## Abstract

**Background/Objectives:** Visual inspection of parenteral drug products is a mandatory and critical unit operation, typically followed by an Acceptable Quality Level (AQL) check, as required by current Good Manufacturing Practices (cGMP) and regulatory authorities worldwide. Visual inspection and AQL checks need to ensure—probabilistically and statistically—that sterile product units with critical, major, or minor defects are excluded from the acceptable portion of a batch, thereby preventing such defective units from reaching distribution and eventually patients. Despite clearly defined batch defect categories, classifying individual defects and assigning them to the correct category remains challenging and has historically lacked standardization and scientific rationale. This paper presents a science-based risk assessment methodology for categorizing defects in sterile dosage forms, incorporating considerations of severity (with emphasis on patient safety), probability of occurrence, and probability of detection. **Methods:** The methodology is based on a modified Failure Mode and Effects Analysis (FMEA), tailored specifically for visual inspection defect classification. **Results:** Three examples demonstrate the practical application of this risk-based approach across different container formats: vials, pre-filled syringes, and cartridges. **Conclusions:** This standardized methodology offers a clear, consistent, and scientifically justified framework for defect classification. Its use enables pharmaceutical manufacturers to establish robust, risk-based defect categorization for the visual inspection of clinical and commercial sterile products.

## 1. Introduction

Visual inspection of injectable drug products (parenteral preparations) is a fundamental quality control measure mandated by pharmacopeial standards, current Good Manufacturing Practices (cGMP), and global health authority expectations [1].

According to cGMP requirements, each container of a sterile drug product has to be visually inspected after filling and closure. During this 100% visual inspection (VI), any units containing visible and detectable defects are identified, removed (culled) from the batch, and rejected. An additional spot check is also often performed after the 100% VI to confirm the suitability of the inspection, being based on a sampling plan with pre-defined acceptable quality limits (AQL), for example, based on ISO 2859-1 or other statistical means [2,3,4]. The various defects can be categorized into critical, major, or minor defects as well as cosmetic imperfections and are associated with different AQL limits per defect category [5]. Process control limits are typically defined based on product manufacturing history, having gained experience with the quantity and type of defects during VI—this includes acceptance criteria for overall yield, as well as acceptance criteria for the maximum amount of defects for each defect category. Such process control limits aim to ensure reliable and robust routine commercial manufacturing.

Table 1 lists an example of defect categories amended by the category cosmetic imperfection, which in some cases remain part of the acceptable part of the batch, including potential definitions, AQLs, and typical ranges, as well as an example for a defect falling into this category.

The detection of defects by visual (manual) inspection is probabilistic and depends on various factors, including inspection conditions such as light type and intensity, whether magnification is used, observation time, inspection background, product parameters including primary packaging material, size and type, fill volume, and product morphology, as well as operator capability and training [6,7,8]. There has hence been quite some debate over the visibility of (visible) particles, depending on size, shape, and type over the past years, i.e., detectability [9].

The 100% VI and AQL checks can be performed manually by trained and qualified operators following the method provided in Ph.Eur. 2.9.20 or USP <790> [10,11]. Although both inspection methods seem highly comparable, they are not identical and not governed for harmonization by the PDG (pharmacopeial discussion group). The non-binding monographs Ph.Eur. 5.17.2 and USP <1790> provide more context from the respective pharmacopeias and outline informational guidance during VI practices for injections, incorporating specific procedural requirements [3,5].

**Table 1:** Example of definition of defect categories of possible AQLs and typical ranges and examples of defect types. Typical AQL ranges as provided * from USP <1790>/ANSI/ASQ Z1.4 or ISO 2859-1 and ** BioPhorum survey [2,4,5,12].

Defect Category	AQL	Definition	Example
Critical	0.040 (typically 0.01–0.1 * or 0.010–0.065 **)	Critical defects can jeopardize the patient’s health, indicate inadequate sterility or non-sealed (non-integral) containers	Crack(s) on the sidewall in the liquid-contact or stopper area
Major	0.65 (typically 0.1–0.65 * or 0.25–1.00 **)	Major defects have no influence on patient health but can change the usability, function, or content of the product	Mobile (non-biological) visible particle with product contact
Minor	2.5 (typically 1.0–4.0 *)	Minor defects have no influence on the product quality but were caused by the production process	Particle(s) or fiber(s) on the needle shield or between the rubber of the needle shield and needle shield without product contact
Cosmetic Imperfections	n/a	Cosmetic/acceptable imperfections represent a solely visual imperfection of the packaging material and have no influence on the product quality; their cause is usually outside the DP production process	Small variation in color on the stopper

VI can also be performed semi-automatically or automatically, especially for large enough batch sizes, and then requires appropriate qualification and cross-validation against the manual method. Some specific “difficult to inspect” products, for example, products that are very turbid, viscous, or filled in very low volume in syringes, may require adaption of the compendial method. Further reading on the topic is provided elsewhere, including Ph.Eur. 5.17.2. [3].

PDA and BioPhorum have evaluated, in a cross-industry survey, how particles are assessed and categorized by pharmaceutical manufacturers and have found significant differences between pharmaceutical manufacturers, lacking consistency in practice, lacking a common rationale for defect classifications, and lacking defect classifications being related to their respective risk [12,13]. BioPhorum has further reported on differences in definitions for defect categories and defect classifications as well as associated AQL limits across pharmaceutical manufacturers [12].

One example related to differences and challenges for an unambiguous defect category assignment is the defect categorization of “visible particles”: the recent BioPhorum cross-industry survey for visible inspection surveying 13 pharmaceutical manufacturers revealed that 38% of companies classify all particles as critical defects, 38% of companies classify all particles as major defects, and 23% of companies split classifications across critical and major defects, likely depending on the type of particle [12].

Given there are many different potential types of defects of a sterile drug product, the lack of controlled studies to evaluate their impact on product CQAs as well as on patient safety is often poorly studied, or just single studies may exist. As an example, the relevance of “stopper ribs” in pre-filled syringes on ensuring container closure integrity (CCI) has not been widely studied despite a recently published study by Pelaez et al. The study revealed that one stopper rib is considered sufficient as a sterile barrier for a pre-filled syringe [14]. Especially for the evaluation of product CQAs on patient safety, these studies are often not available; however, various medical publications may be applied for general considerations (e.g., visible particles and clinical safety) [15,16,17].

In summary, as both the definitions of defect categories, but also the categorization of specific defects by individuals and organizations, may vary, the authors considered it a gap, that there is no relevant science- and risk-based assessment methodology.

### Objective

This manuscript presents a novel, comprehensive, science-based risk assessment methodology for categorizing defects identified during VI of sterile dosage forms. The primary objectives are to

1. Develop a standardized approach for defect classification that incorporates severity considerations, probability of occurrence, and detectability;
2. Demonstrate practical application through examples across different container formats;
3. Provide a framework that aligns with current regulatory expectations while enabling consistent quality decisions;
4. Enable updates as new information and science becomes available.

## 2. Methodology

The presented risk assessment methodology for standardized defect classification in VI of parenteral products builds upon the standard risk assessment and Failure Mode and Effects Analysis (FMEA) methodologies and an adapted approach presented by the Bio-Phorum focus group [12]. In brief, this methodology assigns a numerical risk value to each identified defect in a parenteral biopharmaceutical product. The first step involves defining risk factor categories associated with patient safety (e.g., route of administration, patient characteristics and patient exposure, or particle characteristics). Each risk factor is individually scored and weighted, and the resulting values are multiplied to generate a composite numerical risk score. This score is then used to assign defects to predefined categories—such as critical, major, or minor.

In our approach, we introduced the category of severity to more clearly reflect the potential impact on patient safety, enabling the rapid adoption and adjustment of the assessment based on evolving scientific rationale. Beyond this initial scoring, we incorporated additional elements of FMEA in our approach. In addition to severity, we consider the probability of occurrence and probability of detection (detectability of a given defect during VI). The overall risk factor is calculated as the product of these three parameters (Table 2A), resulting in a final numerical score that determines the classification of a defect into categories such as critical, major, minor, or cosmetic imperfections (Table 2B).

**Table 2:** (**A**) Calculation of risk factors as a product from severity (S), probability of occurrence (O), and probability of detection (D). The risk factor is calculated as the product of these three parameters SxOxD. (**B**) Association of risk factor to defect category.

**(A)**								
**Scoring**	Severity (S)		Scoring	Probability of Occurrence (O)		Scoring	Probability of Detection (D)	
51	Critical defect	Sterility and tightness (CCI) may not be guaranteed Indication of potential microbiological contamination (biological material in the product, e.g., hair/insects) Line clearance or mix-up leading to potential sterility or safety issues Control during filling failed (e.g., 100% fill weight check)	5	Very high	The defect occurs frequently (e.g., in every batch)	5	Very low	The defect is hardly ever detectable
5	High	Seriously ill patients of all ages Intrathecal application More than one dose a day Unusable containers	4	High	The defect occurs repeatedly (e.g., in 1 batch out of 4)	4	Low	The defect is very difficult to detect
3	Moderate	Patients receiving injections or infusions to treat diseases Intravenous, intraocular, and intraarticular application Less than or equal to one dose per day	3	Moderate	The defect occurs occasionally (e.g., in 1 batch out of 15)	3	Moderate	The defect is easy to detect
2	Low	Healthy people without diseases Subcutaneous, intramuscular application One dose a year	2	Low	The defect occurs rarely (e.g., in 1 batch out of 45)	2	High	The defect is reliably detected
1	Very low	No impact on product quality nor patient	1	Very low	The defect never occurs or almost never occurs (e.g., in 1 batch out of 180)	1	Very high	The defect is detected immediately
(**B**)								
**Risk scoring**		**Defect classification**			**Measures**			
≥51		Critical			The defect needs to be sorted out			
>25 and ≤50		Major			The defect needs to be sorted out			
>5 and ≤25		Minor			The defect needs to be sorted out			
≤5		Cosmetic imperfection			Acceptable imperfection and no need to be sorted out			

Furthermore, defects possibly affecting sterility or container closure integrity (CCI) will always be classified as critical due to the inherently high severity score we assigned (i.e., 51) and to avoid potential categorization bias from high detectability. Identified defects for each product configuration are documented in a product-specific defect library or in some cases a generic defect library for platform-based products. These are recommended to include actual pictures or (in lack of actual defect examples) schematic drawings of the described defect to ensure clarity of what the defect encompasses. Defects are then ranked according to the described methodology. Based on their classification, critical, major, and minor defects are subject to rejection, while cosmetic imperfections are monitored but may not have to be rejected depending on company policies.

## 3. Results

The presented science- and risk-based approach for defect categorization for VI was applied to various products along with their various associated potential product defects, employing a range of different primary packaging containers. These included glass and polymer syringes, vials, and cartridges, as well as different fill volumes (including difficult to inspect syringes filled with only 0.1 mL target volume). Here, we present three examples for vials, pre-filled syringes, and cartridge configurations, highlighting the adoption of scientific rationales for the scoring of patient impact (severity), probability of occurrence and detection, and the rapid adoption of the categorization with the most recent scientific advancements. Defects posing a severe risk to patients, such as those potentially affecting sterility or CCI, will always be classified as critical due to the inherently high severity score assigned (i.e., 51). We chose this approach to avoid potential categorization bias arising from high detectability. Using non-linear scales with wider spacing at higher severity levels is a common practice in FMEAs to ensure that severe failures—in our approach and especially those related to patient safety—receive disproportionate weight [18]. This method also confirms that the prioritization produced is sensible in practice. Importantly, it addresses a known limitation of multiplication-based risk score calculations, where very severe but rare events might otherwise appear less urgent than they truly are.

The following case studies demonstrate the capabilities of a more clear assignment of container-specific defects to a given defect category, using the science- and risk-based approach. To note, the assumed probability of occurrence does not reflect the actual incidence within the author’s affiliation.

The classification of visible particles in the solution is generally consistent across different primary packaging systems but may vary depending on detectability and whether a particle may be of “biological origin” (e.g., hair), hence a potential source of microbiological contamination or of “non-biological origin” (e.g., random polyester fiber). In this regard, the aspect of “occurrence” is also important to consider: The random occurrence of single units with single (non-biological) visible particles may be (also clinically) acceptable; however, increased occurrence of those (or other) defects across units (intra-batch) and across batches (inter-batch) warrants further assessment, e.g., via deviations or CAPAs.

The suggested categories introduced in USP <1787> and <1790> [5,19], “**extrinsic” particles** (defined as foreign to the product or manufacturing process), “**intrinsic” particles** (which originate from the formulation or manufacturing process), and “**inherent” particles** (specific to the product itself and represent a characteristic rather than a fault), do not suffice to attribute a defect classification based on risk. Particle identification can support identifying the type of particle, assessing shape, size, and chemical composition. But the same particle may come from different sources (e.g., the container may have been contaminated with a fiber during its production, or the fiber may have been introduced during filling operations). Hence, more specific defect types need to be evaluated. The regulatory implications of this classification approach are discussed in detail in a subsequent section of this manuscript.

### 3.1. Vial Configuration (2R Vial)

Table 3 presents examples of defect classifications for a vial configuration, divided into critical, major, minor, and cosmetic categories, along with their corresponding risk scores. These risk scores were calculated as the product of severity, probability of detection, and probability of occurrence. The probability of occurrence is scored on a scale from 1 (lowest) to 5 (highest) based on the estimated frequency of defects per batch. This parameter provides the most objective basis for risk evaluation, as detailed in the Methods section, since it is directly linked to how often the defect occurs in a batch over a given time period. Critical defects, such as those indicating inadequate sterility assurance or loss of container closure integrity (e.g., non-sealed vials), are assigned the highest severity score by default (51) due to their potential to compromise patient safety and therefore carry the highest overall risk score (see Table 1). In contrast, severity scoring for major and minor defects, also ranging from 1 to 5, requires careful consideration of the current literature and regulatory guidance. In our approach presented in this manuscript, we defined major defects that do not pose a direct risk to patient safety but may impair the usability, function, or content of the product, whereas minor defects have no impact on product quality or patient safety and typically arise from the manufacturing process (see Table 1). As an example, particles detected in the sidewall of the vial, which are in contact with the drug product solution, are rated as a five in severity and categorized as major defect, whereas a fixed particle/fiber on the external side of the container is rated a three (severity) and, overall, as a minor defect.

Cosmetic or acceptable imperfections are limited to visual flaws in the packaging material; these defects do not affect product quality and are unrelated to manufacturing (see Table 1), yet they remain with the batch. Despite their non-impact on quality, such imperfections, but also the other defects, are subject to ongoing monitoring and trending, which may lead to the re-evaluation of their probability of occurrence. If rated higher, this may lead to the re-assessment of the defect category triggering mitigation actions, e.g., a particle in the side wall of the vials will lead to investigation requests at the primary packaging supplier.

The probability of detection depends on several factors, including operator training and experience, the specifics of the VI process, and the nature of the defect, such as particle type and visibility, and has been reviewed and discussed elsewhere [6,7,8,9]. The scoring is higher for difficult to detect defects compared to easy to detect defects. However, the probability of detection may also depend on the individual product configuration and associated fill volume, which leads to differences in scoring between the presented primary packaging configurations. Detection scores are generally informed by prior knowledge (such as industry experience), historical data, and/or scientific evidence, incl. related studies.

### 3.2. Pre-Filled Syringe Configuration (2.25 mL Glass Syringe)

The defect classification for pre-filled syringes follows the same general principles as for vial configurations, particularly regarding missing or incorrectly processed components of the primary packaging container, physical damage (e.g., cracks, scratches), and the presence of particles. However, the risk assessment must be adapted to the specific characteristics of the pre-filled syringe system. This includes the consideration of defects such as particles or fibers on the needle shield or between the rubber component and the outer shield (see Table 4). Certain defects are also specifically associated with container closure integrity (CCI). For example, a critical consideration is whether a defect compromises the sterile barrier of a pre-filled syringe, which is typically defined by the position of the first rib of the stopper, as discussed previously [14].

### 3.3. Cartridge Configuration (10 mL Polymer Cartridge)

For novel container systems such as high-volume cartridges (Table 5), risk categories and defect classifications are generally comparable to those used for conventional containers but must be carefully adjusted to account for defects that are specific or inherent to the new container type. While initial classifications may be informed by knowledge gained during product development and early manufacturing runs, defect libraries are typically updated during commercial batch production as additional experience is accumulated. In such cases, careful evaluation and trending of newly observed defects are essential to ensure appropriate classification and risk assessment.

**Table 3:** Examples of defects and associated risk-based classification for a vial configuration (2R vial). The assumed probability of occurrence does not represent the actual incidence within the author’s affiliation.

Defect Name	Defect Description	Potential Impact	Severity	Probability of Occurrence	Probability of Detection	Risk Score	Defect Classification
Crack(s) on sidewall in liquid or stopper area	Cracks on sidewall, neck, or base, with/ without leakage	CCI, sterility	51	1	2	102	Critical
Vial without stopper	Vial was filled and crimped but no stopper was placed	CCI, sterility	51	1	2	102	Critical
Crimping not tight	Crimping damaged/ dented (tightness of the crimp cap is not guaranteed)	CCI, sterility	51	2	1	102	Critical
Biological-source particle or fiber (hair, insect, etc.) in contact with the product or with the stopper	Particle or fiber of biological origin with contact to the product or to the stopper	CCI, sterility	51	1	2	102	Critical
Particle embedded in sidewall/in contact with solution	Embedded particle in sidewall, neck, or base with contact to solution	Inadvertent non-biological particle	5	4	2	40	Major
Mobile particle/ fiber	Particle or fiber floating in the solution or in vial	Inadvertent non-biological particle	5	4	2	40	Major
Particle/fiber between flip-off cap and crimp cap	Particle or fiber between flip-off cap and crimp cap	Inadvertent non-biological particle	5	2	3	30	Major
Sharp-edged crimping	The crimping is sharp edged but tight (edge not damaged)	Inadvertent issue (usability)	5	2	3	30	Major
Fixed particle/fiber on external side of container	Fixed particle or fiber on external side of sidewall, neck, or base	No impact on product nor patient	3	3	2	18	Minor
Defective or damaged flip-off cap	Flip-off cap is defective or damaged but not broken	No impact on product nor patient	3	2	2	12	Minor
Embedded particle in stopper/no contact with solution	Particle or fiber embedded in stopper	No impact on product nor patient	3	2	2	12	Minor
Discoloration or rough surface of flip-off cap	Discoloration or rough surface of flip-off cap	No impact on product nor patient	1	1	2	2	Cosmetic imperfection
Minor color variation in stopper	Minor color variation of stopper	No impact on product nor patient	1	2	2	4	Cosmetic imperfection
Scratches on flip-off cap	Scratches on flip-off cap (not cracks)	No impact on product nor patient	1	2	1	2	Cosmetic imperfection

**Table 4:** Examples of defects and associated risk-based classifications for a pre-filled syringe configuration (2.25 mL glass syringe). The assumed probability of occurrence does not represent the actual incidence within the author’s affiliation.

Defect Name	Defect Description	Potential Impact	Severity	Probability of Occurrence	Probability of Detection	Risk Score	Defect Classification
Crack(s) on sidewall in liquid or stopper area	Network rupture in glass structure with/without leakage; Crack between 1st rib (incl.) and below needle shield (excluded)	CCI, sterility	51	2	2	204	Critical
Biological-origin particle or fiber (hair, insect, etc.) in contact with the product or with the stopper	Particle or fiber of biological origin with contact with the product or with the stopper	CCI, sterility	51	1	2	102	Critical
Cracked or defective stopper	One or more clear cracks, with/without leakage	CCI, sterility	51	1	2	102	Critical
Puncture, crack, or critical damage of needle shield rubber with impact on CCI	Needle shield rubber damaged and tightness is not given	CCI, sterility	51	1	4	204	Critical
Crack(s) on sidewall between stopper and flange	Network rupture in glass structure without leakage between 1st rib (excl.) and flange (incl.)	Breakage without CCI impact	5	2	3	30	Major
Particle(s) or fiber(s) in 1st rib or on stopper with direct contact with the solution	Particle(s)/fiber(s) in 1st rib with or without direct contact with the solution or on stopper with direct contact with the solution	Inadvertent non-biological particle	3	3	3	27	Major
Particle(s) or fiber(s) on the needle shield or between the needle shield rubber and the needle shield	Particle(s) or fiber(s) present in the needle shield region without contact with the needle/product	No impact on product nor patient	1	2	3	6	Minor
Major variation of color on the stopper	Major color variation in different colors than white and/or gray on the stopper (incl. head, ribs, and trim)	No impact on product nor patient	2	1	3	6	Minor
Embedded or loose particle(s) or fiber(s) on the side of the stopper over several ribs	Particle(s) or fiber(s) at the side of stopper over several ribs but without product contact and not over 1st rib	No impact on product nor patient	3	3	2	18	Minor
Minor damage of needle shield rubber w/o impact on CCI	Needle shield rubber damaged but tightness is still given	No impact on product nor patient	1	1	4	4	Cosmetical imperfection

**Table 5:** Examples of defects and associated risk-based classifications for a cartridge configuration (10 mL polymer cartridge). The assumed probability of occurrence does not represent the actual incidence within the author’s affiliation.

Defect Name	Defect Description	Potential Impact	Severity	Probability of Occurrence	Probability of Detection	Risk Score	Defect Classification
Crack(s) on sidewall in liquid or stopper area	Network rupture in polymer structure without leakage Crack between 1st rib (included) and below tip cap (excluded)	CCI, sterility	51	2	2	204	Critical
Empty cartridge with stopper	Cartridge is empty, but a stopper was placed	CCI, sterility	51	1	1	51	Critical
Cracked or defective stopper	One or more clear cracks, with/without leakage	CCI, sterility	51	2	2	204	Critical
Liquid in 1st ring	Liquid in 1st ring of the stopper	Dose impact	5	2	3	30	Major
Particle embedded in sidewall/contact with solution	Embedded particle with contact with solution	Inadvertent non-biological particle	5	4	2	40	Major
Fixed particle/fiber on septum	Fixed particle or fiber directly on the septum	Inadvertent non-biological particle	5	2	3	30	Major
Crack on flange	Network rupture in polymer structure without leakage, only on flange	No impact on product nor patient	5	1	3	15	Minor
Smear marks on external surface	Smear marks on the external surface of the cartridge	No impact on product nor patient	2	3	3	18	Minor
Particle/fiber in tip cap without contact with the septum	Particle or fiber in tip cap without contact with the septum	No impact on product nor patient	2	5	2	20	Minor
Particle/fiber in 2nd rib (between rib and sidewall)	Particle or fiber on the 2nd rib of the stopper with contact with the sidewall	No impact on product nor patient	2	3	3	18	Minor
Scratches on sidewall or flange	External scratches on sidewall or flange if <0.2 mm wide and <1/3 of body height OR if <0.2 mm wide and circumscribes body <90°	No impact on product nor patient	1	5	1	5	Cosmetic imperfection
Mountain line on tip cap	Mountain line on tip cap	No impact on product nor patient	1	5	1	5	Cosmetic imperfection

## 4. Conclusions

A 100% VI of injectable drug products (parenteral preparations) followed by (statistics- based) AQL checks represents a fundamental quality control measure. During this probabilistic inspection, any unit containing visible and detectable defects are removed from the batch and rejected. The various defects can be categorized into critical, major, or minor defects with different definitions of defect categories and the categorization of defects across pharmaceutical manufacturers.

As there are many different potential types of defects for different product configurations, controlled studies to evaluate their impact on product CQAs, as well as on patient safety, are often not available, or just single studies may exist. In this manuscript, we have reported an advanced science- and risk- based approach for defect categorization for VI building on FMEA analysis and a previously published risk assessment (BioPhorum). By systematically evaluating defects based on patient safety impacts, occurrence probability, and detection capability, this approach provides objective and consistent classification decisions. The three case studies demonstrate the practical application across diverse container formats, revealing both common risk themes and format-specific considerations. The presented examples for vial, pre-filled syringe, and cartridge configurations highlight the adoption of scientific rationales for the scoring of patient impact (severity), probability of occurrence and detection, and the rapid adoption of the categorization with the most recent scientific advancements. This approach demonstrates the capabilities to ensure a more clear, science- and risk-based assignment of container- specific defects to a given defect category.

## 5. Discussion

Visual defects in sterile dosage forms need to be assessed and rejected during 100% visual inspection (VI), which is a key GMP manufacturing unit of operation. Defects can be categorized as critical, major, or minor or defects may be cosmetic (and possibly remaining in the acceptable part of the batch). The categorization of defects is highly relevant, because different defect categories relate to patient safety and product quality differently, as highlighted by different AQL limits, although those are also not standardized across pharmaceutical manufacturers (see Table 1).

Visible inspection is probabilistic in nature, making exact “thresholds” of the visibility for a given defect challenging. This is also acknowledged by requirements as defined by major pharmacopeias. The United States Pharmacopeia (USP) Chapter <1> establishes requirements for visible particulate matter in injections, mandating that products be "essentially free" of visible particles [20]. The Monograph for Parenteral Preparation 0520 of the European Pharmacopoeia (Ph.Eur.) provides similar requirements, emphasizing that parenteral preparations must be "practically free" from particles that can be observed on VI [21]. This terminology acknowledges the practical limitations of achieving absolute particle-free products while maintaining high quality standards [9]. To note, the product appearance and configuration may also impact detectability, as well as operator qualification, inspection conditions (including but not limited to illumination and inspection time) [6,7,8,9].

The assignment of specific defects to a given category has traditionally been based on prior knowledge, experience, and benchmarking. Also, risk-based and science-based in-depth assessments of each defect in multi-page documents may be perceived as challenging to generate and to maintain and may remain arbitrary in their final classification conclusion(s). Accordingly, the assignment of defect categories would benefit from greater standardization grounded in scientific principles and risk assessments. This topic has also been discussed in other forums, such as PDA and BioPhorum, for example, in the context of assigning “visible particles” to a specific defect category [12,13].

### 5.1. Particulate Matter

Particulate matter in injections is defined in USP <1790> [5], as well as by Ph.Eur. Chapter 5.17.2. [3], as “mobile undissolved particles, other than gas bubbles, unintentionally present” in the solutions. Clinical implications of particulate matter in injections are determined by multiple factors and have been reviewed elsewhere [5,18,19,20].

In brief, the "risk to patient" associated with a given defect also depends on the specific characteristics of the injectable product. Key factors include the frequency of administration, the route of administration, and the condition of the patient—for example, whether the patient is immunocompromised. A product containing (non-proteinaceous) particulate matter that is administered intravenously generally poses a greater concern than one administered subcutaneously. To note, a product containing “proteinaceous particles” may be a higher concern for subcutaneous administration (due to potentially increased immunogenicity) than protein particles administered intravenously. Having said that, as taken from Doessegger et al., a product containing single particulates is typically not of a general safety concern [16].

USP <1787> and the informal guideline <1790> have introduced various “categories” for particulates. These are “extrinsic” (“materials that are not part of the formulation, package, or assembly process but rather are foreign and unexpected”, which means being foreign to the manufacturing process deriving from, e.g., the environment, equipment, or personnel), “intrinsic” (“materials … that arise from sources within the formulation ingredients, assembly process, or packaging”, which means coming from within the process, e.g., process residuals or contaminants from production equipment or primary packaging material, formulation including active substances and excipients), and “inherent” (“materials that are expected and thus represent a potentially acceptable characteristic of the product”) [5,18].

Per Ph.Eur., in its recommendation chapter monograph 5.17.2., particles may be “extrinsic” (deriving from the environment, equipment, primary packaging, or personnel), or “intrinsic” (related to the formulation including active substances and excipients, process residuals, or contaminants) [3]. The category of “inherent” was not included into the Ph.Eur. as “any kind of particles in sterile products are unwanted”. The acceptance of “inherent particles” could hence be (mis)used as a waiver to “accept” some kind of defects and defective units that may be mitigated by (time and cost requiring) adequate (formulation) development efforts.

Understanding the specific characteristics of a given defect—such as the nature of particles—is essential for identifying root causes and driving effective corrective and preventive actions (CAPAs) to support continuous improvement. The root cause of defects may stem from various sources, including poor-quality primary packaging components (e.g., contamination introduced during packaging manufacturing, handling, or shipping), formulation-related instability (e.g., protein particles), or unacceptable interactions (e.g., formulation-induced glass leaching or delamination). Contamination during sterile manufacturing processes—such as from environmental sources, mechanical breakage, or other disruptions—must also be considered.

Trending defects and their categorization over time is equally important to support robust quality management. Appropriate mitigation strategies will depend on the identified root cause and may involve collaboration with primary packaging component suppliers to improve quality and establish meaningful specifications. Additional measures may include improvements in manufacturing practices—for example, identifying and eliminating materials (such as specific tissues, wipes, or garments) responsible for particle shedding—or optimizing the formulation to ensure long-term stability and compatibility with both the primary packaging system and materials used during processing. This includes ensuring that the product remains stable and does not, e.g., precipitate throughout its shelf life, thereby meeting regulatory expectations such as being "practically free" of visible particles.

Defect characterization, including particle identification, can be considered a form of forensic science. It is important to acknowledge that visible particle identification cannot be performed on 100% of defective units nor can it detect all defects or particle types. These investigations typically rely on research-grade analytical methods such as flow imaging microscopy, digital microscopy, Raman spectroscopy, FTIR spectroscopy, and other advanced techniques that are not classically validated within a GMP framework. An understanding of the limitations of these methods is essential. Therefore, such defect categorization and particle identification should not be used as the sole basis for batch disposition decisions. However, they can offer valuable insights to support corrective and preventive actions (CAPAs). While it is often possible to distinguish between particle types—such as insect parts, hairs, or fibers—there is a risk of overinterpretation, and conclusions should be drawn with appropriate caution.

In our risk- and science-based approach, we hence did not use the sole categorization of “extrinsic”, “intrinsic”, or “inherent” particles into critical, major, or minor defects given its poor applicability for actual practical use and misaligned approaches in major pharmacopeias. For particles, the approach has been to consider this a major defect, unless the particles are of biological origin (such as hair, insect) that may suggest a potential microbiological concern in addition to the defect and, hence, are considered critical.

### 5.2. Rationale for FMEA Approach

The presented science- and risk-based approach to VI defect classification offers a systematic framework for assessing and categorizing defects based on their potential impact on patient safety, probability of occurrence, and probability of detection.

This methodology supports objective and consistent decision-making by aligning defect severity with product quality attributes (CQAs) and real-world manufacturing conditions. The three case studies illustrate its practical implementation across varying container formats, uncovering both common defect risk themes and format-specific considerations, reinforcing the robustness and adaptability of the method. One of the primary challenges in this area is the diversity and complexity of potential defect types. While some cosmetic or functional defects are well-documented, controlled studies that evaluate their direct impact on product critical quality attributes (CQAs)—and ultimately on patient safety—are often lacking, particularly for subtle or low-frequency defects. In many cases, only isolated studies exist, and for safety-relevant defects, empirical data are sparse or entirely absent. As a result, risk assessments often must draw from the broader medical literature, expert judgment, or analogical reasoning, particularly when dealing with particles. As a result, the risk assessment offers a standardized approach that minimizes uncertainties and supports consistent decision-making.

By incorporating occurrence and detection into the risk evaluation, the framework also accounts for dynamic manufacturing realities. These parameters are sensitive to process variations and can be readily updated in response to observed defect trends. For example, an increase in the occurrence or visibility of a defect triggers a reevaluation of its classification, thereby justifying the need for intensified control or corrective action. In more detail, a defect rated as lower risk initially may be upgraded if its frequency increases, prompting corrective action. Over time, defect histories built from GMP batch evaluations (e.g., initial assessment after 10 batches, followed by reassessment after 30) allow for data-driven control limits and process monitoring. These data, in turn, support the definition of process-specific control limits and facilitate the early detection of deviations from the normal variation. Notably, the structured risk-based classification highlights an industry-wide tendency to over-classify certain cosmetic imperfections—such as minor discoloration or surface scratches. Consistent with insights from industry surveys such as BioPhorum and PDA [12,13], the effective implementation of risk-based classification requires comprehensive product knowledge, mapping of materials, and understanding of particle characteristics.

Overall, the risk-based defect classification strategy offers a scientifically justified method for enhancing VI practices. By incorporating dynamic, data-driven elements such as occurrence and detectability—alongside patient risk—it ensures that inspection programs remain responsive to manufacturing changes, scientifically grounded, and ultimately aligned with patient safety objectives. Notably, the principle reinforces that a risk-based approach is not designed to *avoid* testing, but rather to *optimize* it—directing efforts toward those defects and risks that truly warrant mitigation. Importantly, a risk-based approach does not aim to reduce testing efforts but rather to guide the deployment of adequate, targeted measures—such as analytical identification of particles and specialized test kits—to ensure product quality and patient safety.

### 5.3. Advantages and Limitations

The implementation of a science-based risk assessment approach for defect classification offers significant advantages over traditional arbitrary classification schemes, which vary across pharmaceutical manufacturers. This inconsistency has created challenges for contract manufacturing relationships, technology transfers, regulatory harmonization and supply chain efficiency.

**Objectivity and Consistency**: The structured evaluation process reduces—yet will likely not fully eliminate—subjectivity in classification decisions. Multiple sites or organizations using the same methodology achieve more consistent classifications, facilitating technology transfer and supply chain management. This standardization is particularly valuable for contract manufacturing relationships. 

**Scientific Approach**: Each classification decision is supported by documented risk assessment rationale. This traceability satisfies regulatory expectations for science-based decision-making and provides robust justification during inspections or quality events. The methodology aligns with ICH Q9 principles for quality risk management [22]. 

**Operational Efficiency**: Clear classification criteria enable faster decision-making during batch disposition. Operators understand which defects require documentation and rejection versus documentation only, reducing production holds and quality review cycles. The elimination of unnecessary rejections for cosmetic imperfections improves yield without compromising quality. 

**Continuous Improvement Framework**: The methodology incorporates feedback mechanisms through the periodic reassessment of acceptance criteria. As process understanding improves, classifications can be refined based on actual occurrence data and enhanced detection capabilities. This adaptive approach supports lifecycle management principles. 

While the risk-based methodology represents significant advancements, challenges should be acknowledged. As an example, scoring detection difficulty remains a somewhat subjective standardized criteria (decision matrix), and future research incorporating automated inspection data could refine detection probability assessments. 

It also has to be noted that emerging drug delivery systems may present unique defect types not fully addressed by current categories. In any case, the methodology requires periodic updates to encompass new technologies.

## Data Availability

Data are available on request from the authors.

## Abbreviations

The following abbreviations are used in this manuscript

CAPAs	Corrective and preventive actions
CCI	Container closure integrity
cGMP	Current good manufacturing practice
CQA	Critical quality attribute
FMEA	Failure Mode and Effects Analysis
PDA	Parenteral drug association
Ph.Eur.	European Pharmacopoeia
USP	Unites States Pharmacopoeia
VI	Visual inspection
